# Externalized Keratin 8: A Target at the Interface of Microenvironment and Intracellular Signaling in Colorectal Cancer Cells

**DOI:** 10.3390/cancers10110452

**Published:** 2018-11-16

**Authors:** Marie Alexandra Albaret, Claudine Vermot-Desroches, Arnaud Paré, Jean-Xavier Roca-Martinez, Lucie Malet, Jad Esseily, Laetitia Gerossier, Johan Brière, Nathalie Pion, Virginie Marcel, Frédéric Catez, Geneviève De Souza, Boris Vuillermoz, Franck Doerflinger, Emilie Lavocat, Olivier Subiger, Carine Rousset, Corinne Bresson, Elodie Mandon, Anass Jawhari, Pierre Falson, Mélissa Jasmin, Yohann Coute, Hichem-Claude Mertani, Pierre Saintigny, Jean-Jacques Diaz

**Affiliations:** 1 Univ Lyon, Université Claude Bernard Lyon 1, INSERM U1052, CNRS UMR5286, Centre Léon Bérard, Cancer Research Center of Lyon, 69008 Lyon, France; marie.albaret@lyon.unicancer.fr (M.A.A.); arnopare@gmail.com (A.P.); jxroca@free.fr (J.-X.R.-M.); Lucie.MALET@lyon.unicancer.fr (L.M.); jad_esseily@yahoo.com (J.E.); laetitia.gerossier@wanadoo.fr (L.G.); briere.johan@gmail.com (J.B.); Nathalie.PION@lyon.unicancer.fr (N.P.); Virginie.MARCEL@lyon.unicancer.fr (V.M.); frederic.catez@lyon.unicancer.fr (F.C.); Genevieve.DESOUZA@lyon.unicancer.fr (G.D.S.); MelissaMarie.JASMIN@lyon.unicancer.fr (M.J.); hichem.mertani@lyon.unicancer.fr (H.-C.M.); Pierre.SAINTIGNY@lyon.unicancer.fr (P.S.); 2 Department of Translational Research and Innovation, Centre Léon Bérard, 69373 Lyon, France; 3 iDD Biotech Bâtiment Accinov 317 avenue Jean Jaurès, 69007 Lyon, France; vermotdesroches.claudine@gmail.com (C.V.-D.); vuillermoz.boris@laposte.net (B.V.); franck.doerflinger@gmail.com (F.D.); emilie.lavocat@hotmail.fr (E.L.); Olivier_subiger@yahoo.fr (O.S.); carine.rousset@gmail.com (C.R.); corinnebresson@hotmail.fr (C.B.); 4 Service de Chirurgie Maxillo-Faciale, Centre Hospitalier Universitaire, 37170 Tours, France; 5 CALIXAR Functional Membrane Proteins For Life, 60 avenue Rockefeller, 69008 Lyon, France; emandon@calixar.com (E.M.); ajawhari@calixar.com (A.J.); 6 Laboratory of Molecular Microbiology and Structural Biochemistry, CNRS UMR 5086, IBCP, 69367 Lyon, France; pierre.falson@ibcp.fr; 7 Biosciences and Biotechnology Institute of Grenoble-Laboratoire Biologie à Grande Echelle (BIG-BGE) U1038 INSERM/CEA/UGA, Commissariat à l’Energie Atomique et aux Energies Alternatives (CEA) Grenoble, F-38054 Grenoble CEDEX 9, France; yohann.coute@cea.fr; 8 Department of Medical Oncology, Centre Léon Bérard, 69373 Lyon, France

**Keywords:** *KRAS*-mutant colorectal cancer, externalized keratin 8, therapeutic target, bidirectional signaling, monoclonal antibody

## Abstract

Accumulating evidence supports the remarkable presence at the membrane surface of cancer cells of proteins, which are normally expressed in the intracellular compartment. Although these proteins, referred to as externalized proteins, represent a highly promising source of accessible and druggable targets for cancer therapy, the mechanisms via which they impact cancer biology remain largely unexplored. The aim of this study was to expose an externalized form of cytokeratin 8 (eK8) as a key player of colorectal tumorigenesis and characterize its mode of action. To achieve this, we generated a unique antagonist monoclonal antibody (D-A10 MAb) targeting an eight-amino-acid-long domain of eK8, which enabled us to ascertain the pro-tumoral activity of eK8 in both *KRAS*-mutant and wild-type colorectal cancers (CRC). We showed that this pro-tumoral activity involves a bidirectional eK8-dependent control of caspase-mediated apoptosis in vivo and of the plasminogen-induced invasion process in cellulo. Furthermore, we demonstrated that eK8 is anchored at the plasma membrane supporting this dual function. We, therefore, identified eK8 as an innovative therapeutic target in CRC and provided a unique MAb targeting eK8 that displays anti-neoplastic activities that could be useful to treat CRC, including those harboring *KRAS* mutations.

## 1. Introduction

The unexpected presence of intracellular proteins at the surface of some eukaryotic cells was recently described. These proteins are referred to as externalized proteins (eProts). For most of them, the signals leading to their externalization remain unidentified since, in general, they neither exhibit a canonical transmembrane domain (TM) nor a signal sequence. They appear at the cell surface under pathological circumstances [1], and exhibit new properties related to cell signaling and invasive mechanisms. Indeed, eProts are involved in receptor activation and stabilization, leading to the amplification of signal transduction pathways enhancing cell proliferation. For example, externalized forms of nucleolin or a truncated form of estrogen receptor alpha (ER-α36) are overexpressed in cancer cells and interact with members of the human epidermal growth factor receptor (EGFR) family, providing a positive feedback loop that contributes to enhancing tumor growth [2,3]. Externalized glucose-related protein 94 (eGRP94) interacts with and stabilizes ER-α36 [4], as well as human EGFR 2 (HER2), and induces its dimerization [5]. These cross-talks between eGRP94, ER-α36, and HER2 produce a positive feedback loop in breast cancer [4]. In addition to cell signaling, eProts are also involved in cancer cell invasion. Externalized nuclear protein histone 2B, the externalized endoplasmic-reticulum-associated protein, glucose-related protein 78 (eGRP78), and the externalized cytoplasmic protein, keratin 8 (eK8), are involved in invasive processes through their capacity to act as plasminogen receptors (Plg-receptors) [6,7]. However, at present, the impact of these properties on cancer cell behavior and outcome is understudied.

Keratin 8 (K8) is a constitutive element of the intermediate filament network of many normal epithelial cells. Normal mammary epithelial cells do not express an externalized form of K8 (eK8) [8], while eK8 was reported at the plasma membrane of various carcinomas [9]. Furthermore, eK8 was shown to interfere with the plasminogen/tissue-type plasminogen activator system and to participate in the Plg-dependent activation of matrix metalloproteinases during the invasion process in breast cancer [10]. However, at present, the mechanisms underlying eK8 anchorage at the plasma membrane of cancer cells and its role in tumorigenesis remain to be determined. 

In this study, we demonstrate that eK8 is strongly anchored to the plasma membrane of several types of colorectal cancer (CRC) cells. The predicted topology of this anchoring results in the extracellular exposure of C- and N-terminal domains of eK8, enabling its accessibility via the microenvironment. We generated a unique anti-eK8 monoclonal antibody (MAb), D-A10, determined its eK8-binding domain composed of eight amino acids, and established the proof of concept that this MAb inhibits CRC growth even at low doses. Interestingly, this anti-tumoral effect was observed for both wild-type and *KRAS*-mutated CRCs. We further show that this anti-tumoral effect observed in vivo resulted from the ability of the D-A10 to counteract the control of eK8 over the caspase-dependent apoptotic process. In addition, eK8 stimulated the invasion process of CRC cells in vitro through a plasminogen-dependent mechanism. Altogether, our findings expose eK8 as a key player of colorectal tumorigenesis, the pro-tumoral activity of which can be efficiently inhibited by our in-house generated D-A10 MAb. Therefore, our study highlights eK8 as a promising accessible and actionable therapeutic target for the management of aggressive colorectal tumors, such as those resistant to EGFR inhibition due to their *KRAS*-mutated status.

## 2. Results

### 2.1. Identification of eK8 from a Dynamic Proteomic Analysis

We performed a dynamic proteomic analysis to identify proteins involved in the invasion process of the invasive Isreco-1 CRC cell line. Treatment of Isreco-1 cells with the neuropeptide bombesin (BBS) [11] induced a phenotypic modification resulting in their spreading (Figure S1A,B) and increasing their invasive capacities, which were three fold higher than those of non-treated (NT) cells (Figure S1C). Pulse-labeling with radioactive amino acids was performed on Isreco-1 cells with and without BBS stimulation and was followed by a proteomic analysis. Three proteins displayed the strongest variation in their synthesis upon BBS treatment (red arrows in Figure S1D,E). These proteins were identified by mass spectrometry as three forms of K8 with molecular weights of 54, 45, and 44 kDa, thus revealing that expression and processing of K8 was regulated during the BBS-induced invasion process. Next, detection of K8 was performed by immunofluorescence (IF) with the M20 commercial anti-K8 antibody, on fixed cells permeabilized or not and for cells treated with BBS or cells not treated (NT) (Figure S1F). For non-permeabilized cells, K8 was detected at the surface of the vast majority of NT cells, concentrated in one or two regions of the cell, while it was reorganized and accumulated at the leading edge of BBS-treated invasive cells (white arrow). Following permeabilization, K8 was detected throughout the cytoplasm of both treated and NT cells, as expected for a protein of the intermediate filament network.

### 2.2. An Anti-K8 MAb Panel Exhibits Different eK8 Recognition Properties 

We then generated a panel of M20-like murine anti-K8 antibodies with two immunizing peptides, peptide 1 and peptide 2 (see Supplementary Materials). Among the different MAbs obtained, D-A10 and D-D6 were able to detect full-length K8 (54 kDa) and other forms at 46 kDa, 43 kDa, and over 100 kDa for the D-A10 MAb (Figure S2A) and at 48 kDa and 46 kDa for the D-D6 MAb (Figure S2B). D-A10 and D-D6 MAbs recognized eK8 as shown by IF analyses performed on non-permeabilized Isreco-1 cells (Figure S2C). Specificity of the MAbs was confirmed by Western blot (WB) competition assays with either peptide 1 or 2, or with both peptides at a molecular ratio of 50 or 100 moles of peptide for 1 mole of MAb (Figure S2D,E). Both peptides decreased the detection of all forms of K8 by D-D6, while this was only observed for peptide 1 using D-A10. Recognition by D-A10 MAb was extensively decreased only when peptide 1 was added to peptide 2. These results show that D-A10 and D-D6 MAbs recognize not only common but also different regions of eK8.

### 2.3. Anti-eK8 D-A10 MAb Limits the Plasminogen-Induced Invasion Process

**Figure 1:**
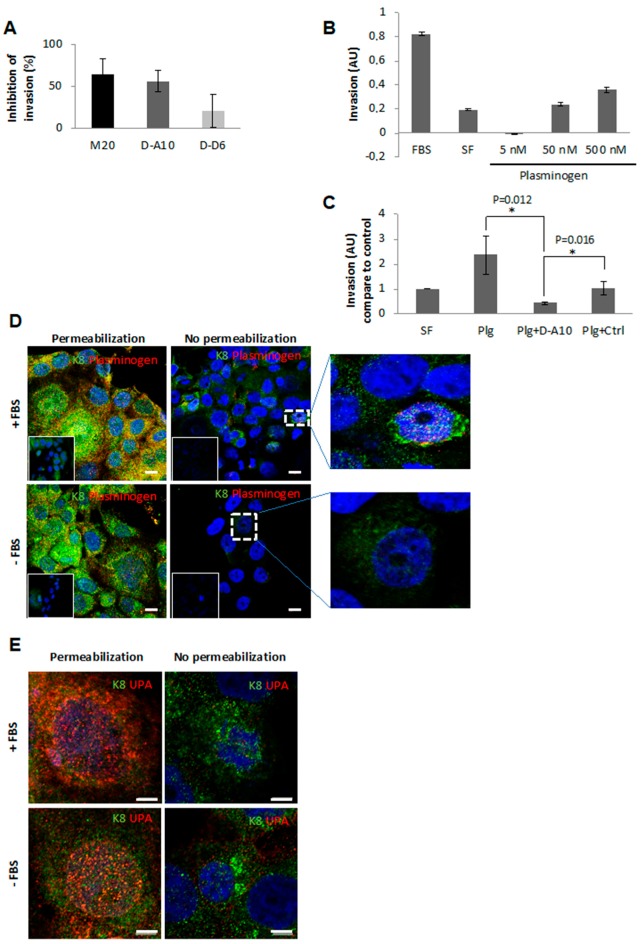
Plasminogen-induced invasion is externalized form of cytokeratin 8 (eK8)-dependent. (**A**) Effect of anti-K8 M20 antibody, and the D-A10 and D-D6 monoclonal antibodies (MAbs) on Isreco-1 cell invasion. Real-time analysis by X-CELLigence (n = 3 ± standard error of the mean (SEM)). Histogram representing the integration of the mean invasion curve slopes (triplicate of three wells per condition ± SEM) over 70 h compared to non-treated cells (% of inhibition of invasion), calculated using the Real Time Cell Analyzer-Dual Purpose, RTCA-DP software^®^ (ACEA Biosciences Inc., San Diego, CA, USA). (**B**) Dose-dependent effect of plasminogen on Isreco-1 cell invasion. Cells were placed for 24 h either in 10% fetal bovine serum (FBS) or in serum-free (SF) medium and resuspended in SF medium containing either no or increasing concentrations of plasminogen (Plg; 5, 50, or 500 nM). Real-time analysis of invasive properties was done using the X-CELLigence technology. Histogram representing the integration of the mean invasion curve slopes (triplicate of three wells per condition ± SEM) over 30 h. AU: Arbitrary Unit. (**C**) Effect of plasminogen in the presence or absence of D-A10 Fab MAb on Isreco-1 cell invasion. Real-time analysis was done using the X-CELLigence system (n = 3 ± SEM). Histogram representing the integration of the mean invasion curve slopes (triplicate of three wells per condition ± SEM) over 30 h compared to control (SF condition). AU: Arbitrary Unit. Student’s *t*-test: * *p* < 0.05. (**D**) Immunofluorescence (IF) analysis by confocal microscopy of K8 (N-terminal antibody, green signal) and plasminogen (anti-Plg antibody, red signal) localization on permeabilized (left panels) and non-permeabilized (right panels) Isreco-1 cells cultured in the presence or absence of FBS. Scale bar = 10 μm. Hoechst dye was used to counterstain the nucleus (blue signal). The inserts represent negative controls without primary antibodies. Superposition or not of green and red signals is presented on enlarged views of the merge images (right panel). (**E**) IF analysis by confocal microscopy of K8 (N-terminal antibody, green signal) and urokinase-type plasminogen activator (uPA; anti-uPA antibody, red signal) localization on permeabilized (left panels) and non-permeabilized (right panels) Isreco-1 cells cultured in the presence or absence of FBS. Scale bar = 5 μm. Hoechst dye was used to counterstain the nucleus (blue signal).

We determined whether MAb neutralization of eK8 could interfere with the plasminogen-induced invasion process. The X-CELLigence analysis of fetal bovine serum (FBS)-stimulated Isreco-1 cell invasion was performed in the presence of 50 µg/mL M20, D-A10, or D-D6 MAbs (Figure 1A). The addition of anti-K8 MAbs induced a decrease in the invasive properties of Isreco-1 cells. M20 and D-A10 MAbs produced a stronger inhibition (64% and 56%, respectively) than that obtained with D-D6.

We then investigated whether inhibition of cell invasion was linked to a competition between MAb and Plg (Figure 1B). The addition of increasing concentrations of Plg to serum-free (SF) medium increased the invasiveness of Isreco-1 cells in a dose-dependent manner. To investigate whether the Plg-induced invasion process could be antagonized by the anti-eK8 D-A10 MAb, we used a Fab fragment of D-A10 MAb lacking its Fc domain that contains a C-terminal lysine (D-A10 Fab MAb) [12] (see Supplementary Materials). This strategy was used to prevent the known binding of Plg with lysine residues at the C-terminal of proteins [6] (see Supplementary Materials). The D-A10 Fab MAb significantly decreased the invasive properties of Isreco-1 cells compared to the control Fab MAb (Figure 1C). Co-localization of eK8 and Plg was observed by IF analyses of Isreco-1 cells in FBS or in SF medium. As shown in Figure 1D and Figure S5A, co-localization of eK8 (green labeling) and Plg (red labeling) was observed in non-permeabilized cells grown with FBS (Figure 1D enlarged view). Urokinase-type plasminogen activator (uPA) required for the conversion of Plg into plasmin [13] was also localized at the plasma membrane of non-permeabilized Isreco-1 cells (Figure 1E—right panels), in the presence or absence of FBS. In addition, intracellular accumulation of uPA (Figure 1E—left panels) under both conditions suggests an autocrine production of uPA by Isreco-1 CRC cells as previously described [11].

### 2.4. Anti-eK8 D-A10 MAb Induces Apoptosis of Colorectal Tumor Cells

**Figure 2:**
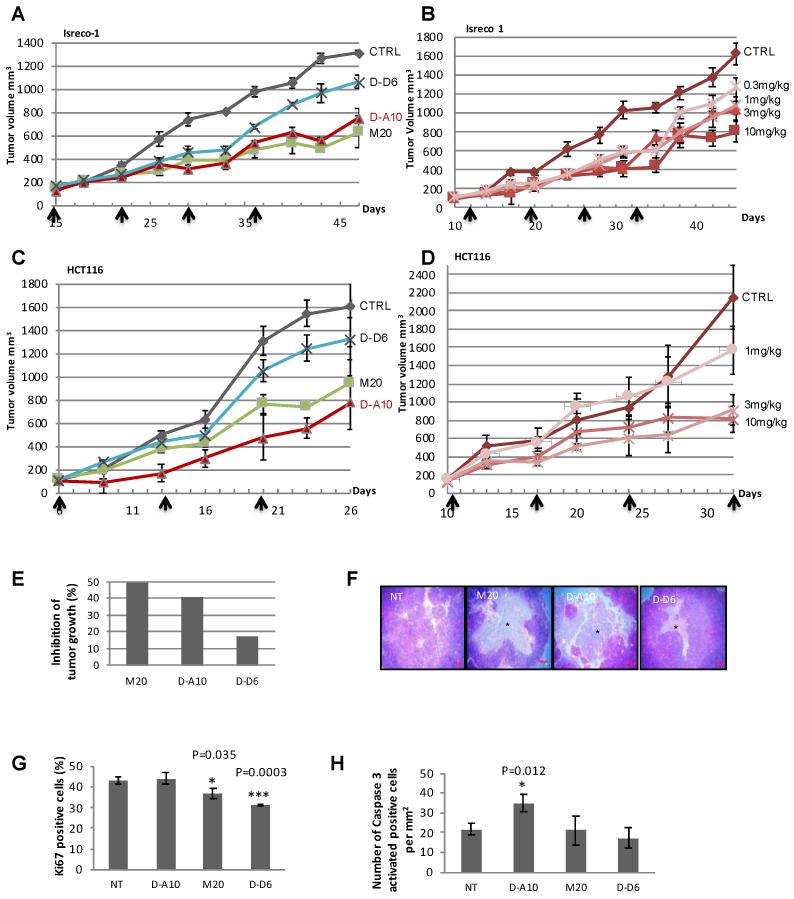
Targeting eK8 using the D-A10 MAb induces apoptosis in vivo. (**A**,**B**) Effect of M20 antibody or of D-A10 and D-D6 MAbs (**A**) or dose-dependent effect of D-A10 MAb (**B**) on Isreco-1 tumor growth. Mouse model of xenograft tumor (three mice per group ± SEM). The average tumor volume of each group of mice is represented on the graph. Antibody intraperitoneal injections performed at the opposite site of the tumor are indicated with black arrows: (**A**)—days 15, 22, 29, and 36; (**B**)—days 12, 19, 26, and 33. (**C**,**D**) Effect of M20 antibody or of D-A10 and D-D6 MAbs (**C**) or dose-dependent effect of D-A10 MAb (**D**) on HCT116 tumor growth. Mouse model of xenograft tumor (three mice per group ± SEM). The average tumor volume of each group of mice is represented on the graph. Antibody injections are indicated with black arrows: (**C**)—days 6, 13, and 20; (**D**)—days 10, 17, 24, and 32. (**E**–**H**) Analysis of the in vivo experiment presented in (**A**). (**E**) Histogram quantifying tumor growth at day 40). Percentage of tumor growth inhibition compared to non-treated mice. (**F**) Immunohistochemical (IHC) analysis by hematoxylin and eosin staining of tumors at the end of the 53-day experimental time course) treated or not with the M20 antibody or with the D-A10 and D-D6 MAbs. (**G**,**H**) Histolab software analysis of the tissue section for quantification of the number of Ki67-positive cells compared to total cells (%) (n = 3) (**G**). Student’s *t*-test: * *p* < 0.05; *** *p* < 0.001. Histolab software analysis of the tissue section for quantification of the number of Casp3A-positive cells (per mm^2^, n = 3) (**H**). Student’s *t*-test: * *p* < 0.05.

We evaluated the effect of eK8 on tumor growth. We established mouse models of colorectal xenograft tumors and treated the mice with M20, D-A10, or D-D6 MAbs (Figure 2). In the Isreco-1 xenograft model, D-A10 MAb strongly decreased tumor growth in a dose-dependent manner and even at low concentrations (3 or 1 mg/kg) (Figure 2A,B). This anti-tumor growth effect was confirmed by analyzing a second model of colorectal xenograft tumor with HCT116 cells (Figure 2C,D). In this model, D-A10 MAb induced the most remarkable anti-tumor growth effect (red curve Figure 2C) even at a low concentration (3 mg/kg) and again through a dose-dependent mechanism (Figure 2D). M20 and D-A10 MAbs induced the strongest anti-tumor growth effects (49% and 40% reduction, respectively) compared to that induced by D-D6 (17% reduction) (Figure 2E). At the end of the treatment (53 days), NT mice developed a large tumor mass with limited necrosis (Figure 2F). Conversely, in mice treated with D-A10 MAb, the tumor mass displayed a dramatic disintegration of the tissue mainly due to the development of large central areas of necrosis which was less prominent in tumors from mice treated with M20 and almost absent in tumors from mice treated with D-D6 MAb.

Cellular proliferation and induction of apoptosis was then evaluated by immunostaining for Ki67 and activated caspase 3 (Casp3A) (Figure 2G,H). Ki67-positive cells decreased significantly in M20- and D-D6-treated mice compared to NT mice, while no difference was observed in D-A10-treated mice. In contrast, the number of activated caspase 3-positive cells was significantly higher in D-A10-treated mice compared to NT mice (Figure 2H). This was not observed in mice treated with the other two antibodies. Taken together, these results demonstrate that D-A10 MAb and M20 exhibit anti-tumor growth effects by inducing a decrease in proliferation (for M20 antibody) or by inducing apoptosis (for the D-A10 MAb).

### 2.5. The LSELEAAL Amino-Acid Motif of eK8 Is Key for Bidirectional Signaling

**Figure 3:**
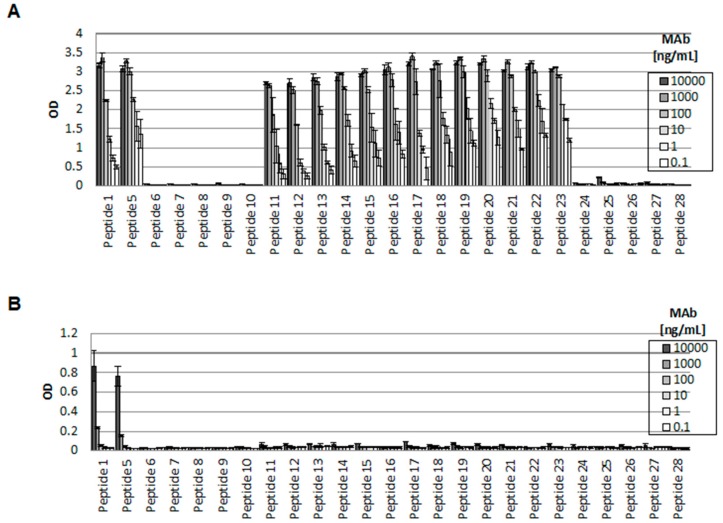
A particular region of K8 is essential for the regulation of invasion and apoptosis. (**A**,**B**) Elisa experiments conducted to determine D-A10 MAb (**A**) or M20 (**B**) reactivity to K8 peptides 1 and 5 to 28. The sequence of each peptide is described in the Table 1. OD: optical density.

We sought to determine the protein sequence of eK8 targeted by the D-A10 MAb. Twenty-four different peptides were produced from immunizing peptides 1 and 2 in order to identify the N- and C-terminal delimitation of the D-A10 MAb epitope. Binding of D-A10 MAb and M20 to the peptides was determined by ELISA (Figure 3 and Table 1). D-A10 bound to peptides 1, 5, and 11 to 23 (Figure 3A), while M20 detected only peptides 1 and 5 (Figure 3B). The leucine at position 353 of peptide 11 was essential for D-A10 binding, since its binding to peptide 10 was almost abolished compared to that to peptide 11. The leucine at position 360 of peptide 23 was also essential for D-A10 binding, since the MAb was unable to bind to peptide 24. In conclusion, the LSELEAAL sequence corresponding to the amino acids 353–360 of the K8 protein sequence constituted the eK8 epitope recognized by D-A10 MAb. 

### 2.6. K8 Is Present at the Plasma Membrane

**Figure 4:**
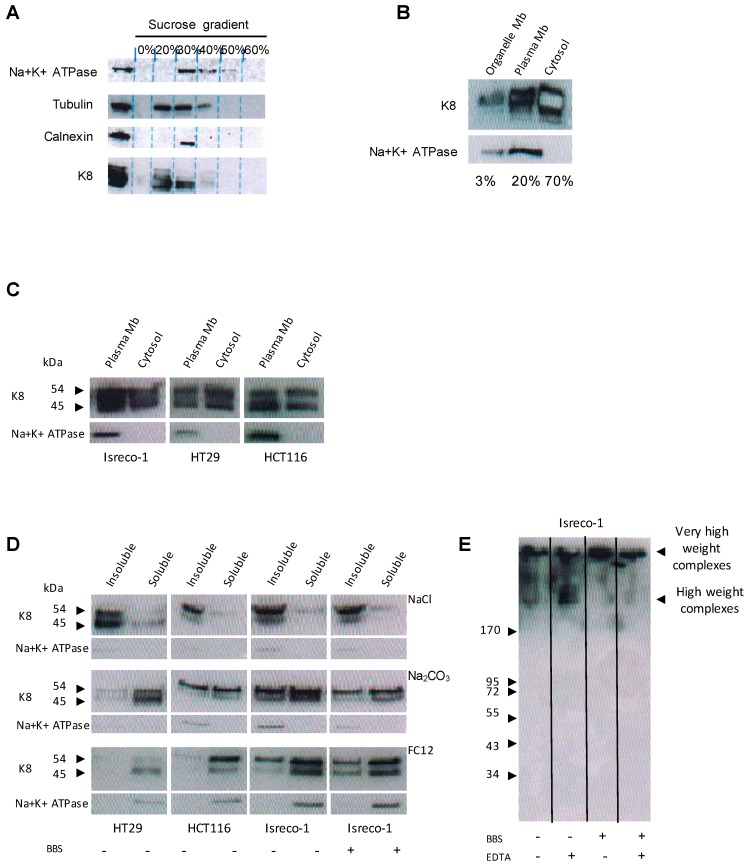
eK8 is a strongly anchored plasma membrane protein. (**A**) Isopycnic centrifugation on sucrose gradient of Isreco-1 cell lysates. Western blot (WB) analysis using the M20 antibody showing that K8 is present in the 20%, 30%, and 40% sucrose fractions and distributed across the cytoplasm, endoplasmic reticulum (ER) membrane, and plasma membrane. (**B**) The separation of cellular components of Isreco-1 cells in order to determine the precise accumulation of K8 in organelle membranes (ER and Golgi), plasma membrane, and cytoplasmic compartments by WB analysis (M20 antibody). (**C**) Plasma membrane enrichment of Isreco-1, HCT116, and HT29 colorectal cancer (CRC) cell lines and WB analysis of K8 accumulation using M20. (**D**) Solubilization of plasma membrane proteins of Isreco-1 (treated or not with bombesin (BBS)), HCT116, and HT29 cells in NaCl, sodium carbonate (Na_2_CO_3_), or FC12 detergent, and WB analysis of K8 accumulation using M20. (**E**) Plasma membrane protein solubilization in the presence or absence of 2 mM ethylenediaminetetraacetic acid (EDTA). The separation of plasma membrane proteins of Isreco-1 cells treated or not with BBS was then conducted on non-denaturing native gels, followed by WB analysis of K8 accumulation using M20.

We characterized the presence of K8 at the plasma membrane using WB coupled to cell fractionation. Subcellular fractions were characterized using Na+K+ATPase, tubulin, and calnexin proteins as markers of plasma membrane, cytoplasmic, and endoplasmic reticulum (ER) membrane fractions, respectively. The most abundant form of K8 was found in cytoplasmic fractions (Figure 4A, fractions 20% to 30% of sucrose). K8 was also present in ER membrane fractions (fraction 30% of sucrose) and in plasma membrane fractions (fractions 30% and 40% of sucrose) (Figure 4A). Relative quantification of K8 distribution between the plasma membrane and membranes from cell organelles and cytoplasm (Figure 4B) revealed that approximately 20% of K8 was present in the plasma membrane, 70% in the cytoplasm, and only a small fraction (about 3%) in other membrane organelles (ER and Golgi). The presence of K8 within plasma membranes was determined for two additional CRC cell lines, HT29 and HCT116 (Figure 4C). 

The biochemical characteristics of K8 anchorage within the plasma membrane of NT HT29, HCT116 and Isreco-1 cells and of Isreco-1 cells treated with BBS were then determined by differential extractions using different types of detergents and salts (Figure 4D). The two major forms of K8 (54 and 45 kDa) were insoluble in NaCl, irrespective of the treatment, but were partially solubilized in sodium carbonate, enabling the solubilization of proteins contained in vesicles. Conversely, both K8 forms were almost completely solubilized by the powerful detergent FC12 (and Triton X100, data not shown), behaving like typical integral membrane proteins, such as Na+K+ATPase. This suggested that K8 was anchored to the plasma membrane. Electrophoretic separation (Figure 4E) was carried out on membrane fractions obtained from Isreco-1 cells treated or not with BBS in non-denaturing conditions in order to identify high-molecular-weight complexes (>170 kDa) in which K8 was accumulated. These high-molecular-weight complexes were not disrupted by the addition of the divalent cation chelator ethylenediaminetetraacetic acid (EDTA), indicating that the formation of K8-containing complexes was independent of divalent cations. Hence, treatment with BBS induces a preferential accumulation of complexes with very high molecular weights. Altogether, these data show that a fraction of K8 was anchored within the plasma membrane of CRC cells in high-molecular-weight complexes. 

### 2.7. N- and C-Terminal Extremities of eK8 Are Exposed at the External Surface of the Plasma Membrane

To further characterize the modalities of eK8 anchoring at the plasma membrane, we analyzed the cellular distribution and the membrane orientation of a full-length GFP-K8 hybrid recombinant protein previously shown to co-localize with the intermediate filament network [14]. In this construct, the GFP sequence was localized at the N-terminal extremity of K8. The expression of the GFP-K8 vector in HCT116 cells was validated by WB with the anti-C-terminal K8 antibodies 1E8 (recognizing the last 13 amino acids of the C-terminus) and M20 at 24 h and 48 h post-transfection (Figure S3A,B).

**Figure 5:**
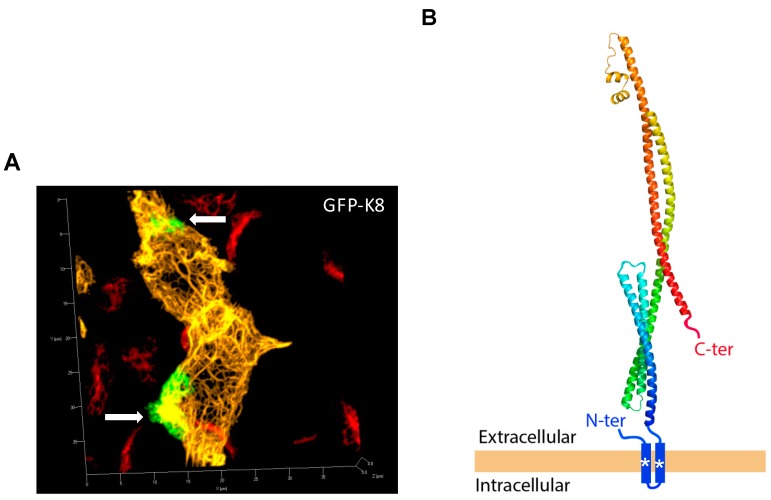
N- and C-terminal parts of eK8 are turned toward the outside of the cell. (**A**) Transfection of HCT116 cells with the GFP-K8 construct and confocal microscopy. Direct visualization of GFP fluorescence (yellow signal) and IF analysis on non-permeabilized cells of GFP (anti-GFP antibody, green signal) and endogenous K8 (M20 antibody, red signal) localization. Three-dimensional (3D) reconstruction of merged images. Externalized GFP-K8 is indicated with white arrows. (**B**) Model of K8 anchorage and topology at the plasma membrane incorporating bioinformatics analyses of K8 structure and transfection experiments of GFP constructs. According to the bioinformatics analysis, alpha helices are represented by different colors (blue, green yellow, and red) and transmembrane domains are indicated with an asterisk (*).

We then performed a thorough determination of the cellular distribution of K8 by IF and confocal microscopy (Figure 5). Fluorescence of GFP fused to the N-terminal part of K8 (Figure 5A yellow color) in transfected cells allowed the visualization of the intracellular intermediate filament network. IF analyses performed on non-permeabilized HCT116 cells with anti-GFP (green color) revealed that GFP-K8 was detectable at the surface of transfected cells mainly within large focalized patches (white arrows). The absence of detection of the intracellular intermediate filament network with the anti-GFP antibody confirmed that the integrity of the plasma membrane was not lost. Importantly, as expected for non-permeabilized cells, eK8 was detected using the M20 antibody and the signal localized within patches at the surface of non-transfected cells (red color) that co-localized with the anti-GFP signal (Supplementary Materials Figure S5C–E). These observations revealed that a fraction of GFP-CK8 was detectable at the surface of cell and was detected with M20 antibody, and that the N-terminal end of CK8 is exposed in the extracellular space. Next, we determined whether the C-terminal part of the hybrid protein was turned toward the outside or inside of the cell. For this, we analyzed the expression of GFP-K8 after transfection of non-permeabilized HCT116 cells by IF using the C-terminal antibody directed against amino acids 420 to 450 (Figure S3C). GFP-K8 was detected using the C-terminal antibody on non-permeabilized cells (white color). Again, as a control of cell membrane integrity, the GFP part of GFP-K8 (green color) highlighted the hybrid protein throughout the intracellular intermediate filament network. These results show that the N- and C-terminal extremities of eK8, anchored to the plasma membrane of HCT116 cells, are exposed to the extracellular matrix.

To refine the topology of eK8 at the plasma membrane, we performed a prediction of the secondary structure of K8 and identified potential non-canonical transmembrane (TM) domains. We identified proteins exhibiting local similarities with K8 using the Protein Homology/analogy Recognition Engine V 2.0 (Pyre2) software. These analyses revealed a 99.6% sequence homology with the transmembrane protein colicin-ia for a coverage of 79%. Homology with colicin-ia confirmed the helical coiled-coil structure of K8 (Figure S4A). We then determined the predicted TM domains of K8 using the TMHMM Server v.2.0 and TMPRED software. K8 was predicted to contain one TM domain in its N-terminal region (surrounding amino acids 45 to 75) according to TMHMM (Figure S4B) and TMPRED (data not shown). The primary sequence of the N-terminal region of K8 contains two hydrophobic regions probably organized in non-canonical alpha helices (amino acids 45 to 63 and 68 to 88) that could be sufficient for its anchorage within the plasma membrane (Figure S4C). These analyses combined with the IF analyses enabled us to propose a model in which K8 could contain several alpha helices and could be anchored to the plasma membrane via a TM domain localized in the N-terminal region of K8 (Figure 5B). Of note, both C- and N-terminal extremities of eK8 are on the extracellular surface of the cell.

## 3. Discussion

In this study, we unraveled a novel function in colorectal tumors of the externalized form of K8 that we named eK8. We showed that eK8 is anchored within the plasma membrane. We generated a novel anti-eCK8 MAb, D-A10, directed against an eight-amino-acid-long epitope of eK8. Using the D-A10 MAb, we demonstrated that eK8 mediates bidirectional signaling from colorectal tumors, one to the microenvironment leading to extracellular matrix degradation and another to the intracellular apoptotic machinery leading to tumoral cell death in vivo. 

We determined the nature of eK8 anchorage, which has so far not been investigated [15,16]. Analysis of K8 distribution across different cellular compartments showed that about 20% of the total cellular amount of K8 was present at the plasma membrane. Extraction procedures using different types of detergents enabled us to demonstrate that eK8 was anchored to the plasma membrane of several CRC cell lines, including the *KRAS*-mutated Isreco-1 and HCT116 cell lines. Using IF performed on non-permeabilized cells with a panel of anti-K8 MAbs, as well as GFP-K8 hybrid proteins, we showed that both N- and C-terminal extremities of eK8 were exposed at the exterior of CRC cells. K8 domains localized at the C-terminal region containing amino acids 353 to 360 (D-A10 MAb), 353 to 367 (M20 antibody) [17], and 420 to 450 (C ter MAb) were exposed at the exterior of CRC cells. This finding was complementary to previous data showing that the C-terminus of eK8 encompassing the last 13 amino acids is exposed to the extracellular space of breast cancer cells [12]. In addition, GFP-K8 hybrid proteins, in which the GFP portion was localized at the N-terminal end of K8, coupled with IF analyses, revealed that the N-terminal extremity of eK8 was also present on the outer leaflet of CRC cells.

Nevertheless, the dozen of well-known externalized proteins described in the literature, including K8, are often characterized by the absence of canonical TM and signal peptides [9]. Notably, K8 was previously described at the cell surface as a plasminogen receptor while its amino-acid sequence lacks a transmembrane portion [18]. We used prediction software (TMHMM) [19] to predict TMs. We also used another bioinformatic tool (Phyr2) [20] to predict the overall structural topology of K8, which suggested two TM domains and a large extracellular domain. Indeed, secondary-structure prediction strongly suggested that anchorage within the plasma membrane was due to a yet unidentified TM in the N-terminal region of K8. eK8 anchorage could be due to two non-canonical alpha helices encompassing residues 45 to 63 and 68 to 88. All of the residues of these domains, except residues 79 and 81 (D and E that are negatively charged) are hydrophobic and resulted in their positioning within a typical plasma membrane biochemical environment. One of the two alpha helices (amino acids 45 to 63) is particularly enriched in glycine. This particular composition provides a biophysical plasticity that may account for a transitory structural flexibility. Solid-state nuclear magnetic resonance spectroscopy previously established that the glycine/serine-rich end domains of mouse epidermal keratin intermediate filaments have little or no structural stabilized order [21]. These domains which lack three-dimensional (3D) structure are found in proteins with intrinsically disordered regions (IDRs) and provide inherent flexibility to these proteins that can adopt several conformations in solution [22]. Therefore, the repetition of glycine in the N-terminal region of K8 may constitute one of these IDRs and confer plastic properties to K8. Of note, these domains are different from one cytokeratin to another and, therefore, may provide each cytokeratin with specific biophysical properties [23]. The possibility that eK8 anchorage into the plasma membrane was due to these two non-canonical alpha helices was reinforced by our demonstration that the presence of divalent cations was not necessary for eK8 anchorage, since divalent cations (especially calcium) may allow the direct association of extrinsic proteins to the phospholipid head of membrane lipids independently of the presence of a well-structured hydrophobic domain. Altogether, these experiments coupled to in silico analyses led us to propose a model in which eK8 N- and C-terminal regions were exposed at the external part of CRC cells, while anchorage within the plasma membrane was due to a transmembrane domain in a highly flexible region of K8 containing two non-canonical alpha helices. Here, we predict a model that describes the overall structural topology of this key protein providing structural information to combine with experimental data. Such a model cannot include information about conformational states of functional relevance. Further structural work is required to gain insights into structure–function relationships of K8 alone and in complex with potential partners such as type I keratin, like K18. Using confocal microscopy and Western blot analysis with native gels, we showed that eK8 could form large oligomers at the cell surface even in the absence of divalent cations. This property may be necessary for eK8 to exist in a state of oligomerization in order to support plasminogen activation [10]. Interestingly, our observation that bombesin treatment of Isreco-1 cells induces the formation of very-high-molecular-weight complexes of eK8 supports this possibility of an eK8/plasminogen complex formation. The unusual presence at the cell surface of supra-molecular structures made of proteins that are usually found intracellularly was also described for histones that could be assembled as nucleosomes anchored to the cell surface through an interaction with sulfated polysaccharides. However, the function of the cell-surface nucleosomes remains unclear [24].

Although we did not elucidate the molecular mechanism via which eK8 could be exported to the exterior of the plasma membrane, several hypotheses could be considered. Firstly, the translocation of eK8 to the plasma membrane could be mediated by ATP-binding cassette (ABC) transporters, since these ABC transporters, the main family of ATP-activated pumps, were characterized as transporters with a large specificity for various substrates such as inorganic ions, amino acids, peptides, or proteins, as well as lipid transporters [25]. eK8 may be externalized by one of them, e.g., a chloride transporter such as the cystic fibrosis transmembrane regulator, since a global proteomic approach showed that the intracellular distribution of K8/K18 could be modulated by this transporter [26]. Secondly, exocytosis by exosomes or thirdly, transleaflet lipid movement (flip-flop) could also be proposed as mechanisms of eK8 translocation, though this merits further investigation.

In order to decipher more finely the function of eK8, we used a K8-targeting strategy based on our in-house designed anti-K8 MAbs. Several anti-K8 MAbs able to interfere with K8 metabolism or intracellular functions were described in the literature [16,27,28,29,30]. However, none of them were identified and fully characterized as MAbs interfering with eK8 functions. Among these antibodies, we selected the commercially available anti-K8 antibody M20, the monoclonal nature of which was uncharacterized, to generate an anti-CK8 MAb panel. We designed two immunogenic peptides encompassing the region of K8 recognized by the M20 antibody [17] and generated a panel of anti-K8 MAbs. D-D6 and D-A10 were able to detect the most abundant K8 forms at 54 kDa and 46 kDa present in the plasma membrane, and eK8 at the cell surface. The D-A10 MAb detected additional K8 forms and, notably, two high-molecular-mass isoforms (>100 kDa) that could be K8 oligomers. Only one, D-A10 MAb, strongly inhibited the invasive FBS, as well as Plg-induced process. The Plg-induced invasion process was dose-dependent and strongly inhibited by the addition of a Fab fragment of the D-A10 MAb. Using IF on non-permeabilized cells, we detected Plg and uPA at the cell surface, and we observed the co-localization of Plg with K8. This strongly suggested that the invasion process induced by FBS and Plg was, at least in part, due to the activation of the Plg–uPA system via its interaction with eK8.

Altogether, these results showed that eK8 provides CRC cells with the intrinsic capacity to respond to Plg activation as previously shown in breast cancer cells [15,31]. The Plg system is involved in cell invasion, in metastasis formation, and in tumor growth [18]. Moreover, it was suggested that eK8 in complex with uPA and plasminogen was able to modulate in cellulo cell adhesion and proliferation [32]. Therefore, we then determined the impact of our two anti-eK8 MAbs D-A10 and D-D6 on tumor growth in vivo.

Using xenografted mice bearing Isreco-1 or HCT116 tumors treated with different doses of M20, D-A10, or D-D6 MAb, we showed that the M20 antibody, as well as the D-A10 MAb, induced a continuous and significant reduction in tumor growth, while the D-D6 MAb did not produce this effect. Strikingly, histological staining of the Isreco-1 tumors demonstrated that a large necrotic area in M20-treated tumors also observed in D-A10-MAb-treated tumors is accompanied by tissue disintegration. In order to decipher the molecular mechanisms via which M20 and D-A10 MAb limited the growth of Isreco-1 tumors, we performed immunohistochemistry (IHC) staining and quantification of Ki67 and Casp3A, which revealed that D-A10 and M20 exhibited distinct anti-tumor growth effects. Indeed, D-A10 MAb was the only MAb tested to induce an important increase in Casp3A signaling, demonstrating that its anti-tumor growth activity was associated with caspase-dependent apoptosis. Conversely, D-D6 MAb induced a reduction in the Ki67 index, but no anti-tumor growth activity was observed. There is accumulating evidence that K8/K18 could also exert non-mechanical functions providing cells with resistance toward apoptotic stress or inversely favoring apoptosis. Thus, it was proposed that K8 phosphorylated on residue Ser 73 may act during stress as a “phosphate sponge”, absorbing the stress-activated protein kinases (SAPK), thereby reducing their untoward effects and limiting the induction of apoptosis. K8 S73 is a unique and readily available SAPK substrate because it behaves as a switch that is either “on” or “off”, being completely dephosphorylated (off) under basal conditions, but being turned on via phosphorylation during apoptosis and cell injury [33]. It was demonstrated that the ubiquitous death effector domain containing the DNA-binding protein (DEDD) could bind intracellular K8/K18 matrix and also caspase 9 and caspase 3 [34]. Consequently, it was suggested that K8/K18 intermediate filaments may provide a scaffold for proximity-induced auto-cleavage and activation of caspase [35].

Finally, by identifying the LSELEAAL motif as the epitope of D-A10 MAb, we demonstrated that the bidirectional signaling of eK8 is mediated by a motif localized at the C-terminal region of the protein exposed at the exterior of the CRC cells. Several anti-K8 antibodies were described with a capacity to interfere with specific functions of K8. For example, two MAbs directed against the VKIALEVEIATY K8 motif led to unveiling the role of K8 in the plasminogen–uPA system in breast cancer [15,31,32,36]. However, none of these MAbs were directed against the LSELEAAL motif of K8 and were, therefore, not able to unravel its bifunctional properties.

Overall, our results obtained in vivo and in vitro reveal a novel and unexpected function for eK8; eK8 promotes invasion through activation of the Plg system via direct binding and protects cells from apoptosis. These two properties of eK8 could represent key molecular mechanisms leading to the formation of metastases, making eK8 a relevant target in colorectal cancer, including *KRAS*-mutant diseases that are associated with resistance to EGFR inhibition.

Our study illustrates that eProts exhibit functions that appear to be much more complex than previously anticipated and, as such, represent an important reservoir of fascinating targets that should be evaluated in the near future.

## 4. Materials and Methods

### 4.1. Cell Lines

The Isreco-1 colorectal cancer (CRC) human cell line was a gift from Pr. J.-C. Saurin and Dr. J. Abello (Lyon, France). HCT116 and HT29 colorectal cancer (CRC) human cell lines were supplied by the American Type Culture Collection (ATCC) and Deutsche Sammlung von Mikroorganismen und Zellkulturen (DSMZ), respectively.

### 4.2. Antibodies

The different antibodies used were mouse monoclonal anti-K8 antibody (clone M20, Sigma-Aldrich, Darmstadt, Germany), mouse monoclonal anti-C terminal K8 antibody (clone 1E8, Abcam, Cambridge, UK), rabbit polyclonal anti-C terminal K8 antibody (SAB4501654, Sigma-Aldrich, Darmstadt, Germany), rabbit polyclonal anti-N terminal K8 antibody (ab137855, Abcam, Cambridge, UK), proprietary mouse monoclonal anti-K8 antibodies (clone D-A10 and clone D-D6), mouse monoclonal anti-uPA antibody (ab131433, Abcam, Cambridge, UK), mouse monoclonal anti-plasminogen antibody (ab38157 Abcam, Cambridge, UK), rabbit polyclonal anti-caspase 3 cleaved (Asp 175) antibody for IF analysis (9661, Cell Signaling Technology, Leiden, The Netherlands), rabbit monoclonal anti-cleaved caspase 3 antibody for IHC analysis (clone 5A1E, Cell Signaling Technology, Leiden, The Netherlands), mouse monoclonal anti-caspase 3 and cleaved caspase 3 Alexis antibody for Western blot (WB) analysis (Enzo Life Science, Farmingdale, NY, USA), mouse monoclonal anti-Ku80 antibody (ab3715, Abcam, Cambridge, UK), mouse monoclonal anti-histone H3 antibody (ab1791, Abcam, Cambridge, UK), anti-Na+/K+ ATPase alpha (H3) (SC-48345, Santa Cruz Biotechnology, Santa Cruz, CA, USA) rabbit polyclonal anti-GFP antibody (ab290, Abcam, Cambridge, UK), and mouse monoclonal anti-Ki67 antibody (Clone Mib1, M7240 DAKO, Santa Clara, CA, USA).

### 4.3. Cell Fractionation Procedures

For cytoplasm–nucleus fractionation, the protocol was performed as previously described [37].

For the separation of cellular components, lysis buffer (10 mM Tris-HCl, pH 8.0) was added to cells which were kept on ice for 1 h. The cells were then lysed using a mechanical Dounce homogenizer. Then, 250 μL of lysate was placed at the top of a gradient composed of sucrose layers (20–60%) with increasing density. Next, an isopycnic centrifugation at 200,000× *g* for 15 h led to the separation of cellular components.

For plasma membrane enrichment, lysis buffer (10 mM Tris-HCl, pH 8.0) was added to cells which were kept on ice for 1 h. The cells were then lysed mechanically using a Dounce homogenizer. The lysate was centrifuged at 500× *g* for 10 min in order to remove intact cells and cell debris. The supernatant was then centrifuged at 15,000× *g* for 30 min to recover the organelle membranes (Golgi, endoplasmic reticulum). Plasma membranes were collected by centrifugation of the supernatant at 100,000× *g* for 1 h. The cytoplasmic proteins were contained in the remaining supernatant. Finally, 500 μL of storage buffer was added to the different pellets (20 mM Tris-HCl, pH 8.0, 50 mM NaCl, 20% glycerol, 1 mM EDTA, 1× protease inhibitor).

For membrane anchoring analysis, plasma membranes were diluted at a concentration of 1 mg/mL in solubilization buffer (20 mM Tris-HCl, 100 mM NaCl, final volume of 100 μL) supplemented with either NaCl (1 M), sodium carbonate (100 mM Na_2_CO_3_, pH 11.5), or detergent FC12 (1%). The solubilization of plasma membranes in the presence or absence of 2 mM EDTA was also tested. Solubilization was performed at room temperature for 1 h, and the solubilized fractions and insoluble fractions were subsequently separated by centrifugation at 100,000× *g*.

### 4.4. Immunofluorescence Analysis

If necessary, glass coverslips were pre-coated with a solution of matrigel (Corning, New York, NY, USA) in culture medium at a dilution of 1:20 for 30 min at room temperature (for BBS-induced invasion or co-localization of K8 with uPA or plasminogen experiments). To preserve membrane-associated components, non-permeabilized cells were fixed in 4% paraformaldehyde (PFA) at room temperature (RT) for 5 min. In contrast, permeabilized cells were fixed in 4% PFA at RT for 30 min. Membrane permeabilization was conducted by adding Triton X-100 (Sigma-Aldrich, Darmstadt, Germany) for 5 min. For protein expression analysis at the plasma membrane, a solution of phosphate-buffered saline (PBS) containing primary antibodies was incubated for 2 h with glass coverslips. For intracellular protein expression analysis, primary antibodies were added to a PBS solution containing 0.1 M bovine serum albumin (BSA), 0.3 M NaCl, 0.5% Tween-20, and 1% FBS, and then incubated with glass coverslips for 1 h at RT.

Primary antibodies used were as follows: anti-K8 M20 (20 μg/mL), D-A10 (78 μg/mL), D-D6 (78 μg/mL), anti-C terminal K8 (20 μg/mL), anti-N terminal K8 (1:250), anti-GFP (1:500), anti-uPA (1:100), or anti-plasminogen (1:100). Primary antibodies were detected using Alexa-488 or Alexa-555 secondary antibodies coupled to fluorophores for 1 h at RT. Cells then were incubated in a PBS solution containing Hoechst dye (Sigma-Aldrich, Darmstadt, Germany) diluted 1:10,000 in order to counterstain cell nuclei.

## 5. Conclusions

In the present study, we showed for the first time that the externalized form of K8, eK8, can induce an anti-apoptotic signal in addition to its already known ability to promote an invasive plasminogen-mediated signal. This bidirectional signaling of eK8 provides a new function that could be key for CRC tumor progression, particularly for metastasis formation.

## Figures and Tables

**Table 1:** Number and sequence of keratin 8 (K8) peptides targeted by D-A10 and M20 monoclonal antibodies (MAbs).

Peptide (N°)	Sequence K8 (aa)	Antibody Reactivity on Coated Free Peptides	
		D-A10	M20
**Peptide 1**	338-AEQRGELAIKDANAKLSELEAALQRAKQD-C-366	+++	+
**Peptide 5**	338-AEQRGELAIKDANAKLSELEAALQRAKQD-366	+++	+
**Peptide 6**	358-AALQRAKQD-366	-	-
**Peptide 7**	357-EAALQRAKQD-366	-	-
**Peptide 8**	356-LEAALQRAKQD-366	-	-
**Peptide 9**	355-ELEAALQRAKQD-366	-	-
**Peptide 10**	354-SELEAALQRAKQD-366	-	-
**Peptide 11**	353-LSELEAALQRAKQD-366	++	-
**Peptide 12**	352-KLSELEAALQRAKQD-366	++	-
**Peptide 13**	351-AKLSELEAALQRAKQD-366	++	-
**Peptide 14**	350-NAKLSELEAALQRAKQD-366	+++	-
**Peptide 15**	349-ANAKLSELEAALQRAKQD-366	+++	-
**Peptide 16**	348-DANAKLSELEAALQRAKQD-366	+++	-
**Peptide 17**	345-C-AIKDANAKLSELEAALQRAKQD-366	+++	-
**Peptide 18**	345-AIKDANAKLSELEAALQRAKQ-365	+++	-
**Peptide 19**	345-AIKDANAKLSELEAALQRAK-364	+++	-
**Peptide 20**	345-AIKDANAKLSELEAALQRA-363	+++	-
**Peptide 21**	345-AIKDANAKLSELEAALQR-362	+++	-
**Peptide 22**	345-AIKDANAKLSELEAALQ-361	+++	-
**Peptide 23**	345-AIKDANAKLSELEAAL-360	+++	-
**Peptide 24**	345-AIKDANAKLSELEAA-359	-	-
**Peptide 25**	345-AIKDANAKLSELEA-358	-	-
**Peptide 26**	345-AIKDANAKLSELE-357	-	-
**Peptide 27**	345-AIKDANAKLSEL-356	-	-
**Peptide 28**	345-AIKDANAKLSE-355	-	-

The levels of M20 antibody and D-A10 MAb reactivity to recognize peptides are indicated with the following signs: +++ (strong), ++ (medium), + (weak), or - (none).

## Supplementary Materials

The following are available online at http://www.mdpi.com/2072-6694/10/11/452/s1, Figure S1: K8 is accumulated in a cellular model of colorectal cancer cell invasion; Figure S2: Characterization of D-A10 and D-D6 MAb properties; Figure S3: Expression of GFP-K8 constructs; Figure S4: Analysis of K8 secondary structure and primary sequence; Figure S5: Confocal analysis of K8–Plg and K8–GFP co-localization by immunofluorescence (IF).
