# Supplementary material for: Externalized Keratin 8: A Target at the Interface of Microenvironment and Intracellular Signaling in Colorectal Cancer Cells
Source: Cancers (Basel). 2018 Nov 16;10(11):452. doi: 10.3390/cancers10110452 (PMC6266717; doi:10.3390/cancers10110452)
Supplement: Supplementary file 1 [file cancers-10-00452-s001.pdf]

cancers

MDPI

Supplemental Materials and Methods: Externalized keratin 8: a target at the interface of microenvironment and intracellular signaling in colorectal cancer cells

Marie Alexandra Albaret, Claudine Vermot-Desroches, Arnaud Paré, Jean-Xavier Roca-Martinez, Lucie Malet, Jad Essely, Laetitia Gerossier, Johan Brière, Nathalie Pion, Virginie Marcel, Frédéric Catez, Geneviève De Souza, Boris Vuillermoz, Franck Doerflinger, Emilie Lavocat, Olivier Subiger, Carine Rousset, Corinne Bresson, Elodie Mandon, Anass Jawhari, Pierre Falson, Mélissa Jasmin, Yohann Coute, Hichem-Claude Mertani, Pierre Saintigny, Jean-Jacques Diaz

Cell culture and treatments. Isreco-1, HCT116 or HT29 colorectal cell lines were grown in Dulbecco's Modified Eagle's Medium (Sigma, St Quentin Fallavier, France) supplemented with 10% heat-inactivated fetal bovine serum (FBS) (Sigma, St Quentin Fallavier, France), 4 nM L-glutamine (Sigma, St Quentin Fallavier, France) and 50 U/mL, 50 µg/mL penicillin -streptomycin (Sigma, St Quentin Fallavier, France).

Bombesine (BBS) treatment: cells were seeded in a culture medium containing 0.1% bovine serum albumin (BSA) and 1% of penicillin-streptomycin. 48 h later, culture medium was renewed and BBS was added at a final concentration of 10 nM.

D-A10 MAb treatment: A solution of D-A10 MAb at 1 µg/mL in complete cell culture medium was added to cells for a treatment of a minimum of 24 h and a maximum of 140 h.

Plasminogen treatment: A solution of active full length native human plasminogen purified from human plasma (Abcam) at 5, 50 or 500 nM in serum-free culture medium was added to cells for 75 h.

Preparation of anti-K8 MAbs. Murine monoclonal antibodies specific for K8 were produced using standard hybridoma techniques (Johnson, McNamara et al. 1981). Two different K8-related peptides (peptide 1 and peptide 2) were chosen within the M20 antibody recognized portion of K8 (Van Muijen, Ruiter et al. 1987 and Waseem, Karsten et al. 2004), synthesized and used for mice immunization. The peptide sequences are as follows:

Peptide 1: AEQRGELAIKDANAKLSELEAALQRAKQD-C

Peptide 2: AEQRGELAIKDANAKLSELE-C

Immunization was performed on three OF1 mice with a mixture of peptides 1 and 2 coupled to KLH. The spleens of mice whose serum was positive for K8 by ELISA were selected. The splenocytes were fused with mouse myeloma line X63-Ag8.653. Hybridoma supernatants were screened for K8 binding by ELISA and by immunofluorescence cell staining on K8-positive cell lines. After hybridoma cloning, two murine MAbs were obtained called mD-A10 and mD-D6. The D-A10 or D-D6 clones were injected into the peritoneum of nude mice. The ascitic fluid was purified by affinity chromatography on protein A column and then IgG was eluted at an acidic pH and then transferred to PBS. After concentration, the PBS solution containing IgG was filtered and the MAb concentration was determined at 280 nm.

Preparation of Fab fragments. Solutions of IgG1 at 5 mg/ml were prepared in buffer containing 5 mM EDTA (Sigma Aldrich, ref. 03677), 5 mM L-Cysteine (Sigma Aldrich, ref. C7352) with papain (Sigma Aldrich, ref. P4762) dissolved in PBS 1X buffer (Gibco, ref. 20012019) to give an enzyme to antibody ratio of 1:100 (w/w). The resulting solutions were incubated for 3 h, 4 h or 5 h (according to the optimal digestion time of antibodies) at 37°C in a water bath. To stop the digestion, 50 mM iodoacetamide (Sigma-Aldrich, ref. I1149) was added and the reaction mixtures left for 30 m at room temperature (RT). Digested samples were injected onto a Superdex75 10/300 GL column (GE Healthcare, ref. 17-5174-01) at a flow rate of 0.5 mL/min and fractions of interest (Fab and Fc

fragments) were collected. Separation of the fragments was accomplished using a protein A column (GE Healthcare, Hi Trap MAbSelect Sure 1 ml ref. 11-0034-93). The Fab fragments were checked for purity by High Performance Size Exclusion Chromatographic (HPSEC) analysis and sodium dodecyl sulfate-polyacrylamide gel electrophoresis (SDS-PAGE).

Radioactive labelling. Staining was conducted for 1 h before the harvesting of cells. For that purpose, we replaced DMEM cell culture medium without methionine and cysteine but complemented with methionine and cysteine tagged with sulphur S^{35} at the rate of 100 µCi/ mL. Cells were then incubated for 1 h at 37°C under 5% CO_2.

Protein separation in 2D electrophoresis. Separation of proteins in 2D electrophoresis was performed as described previously (Belin, Beghin et al., 2009).

Isoelectrofocalisation: 1.5 mg of protein in rehydration buffer (8 M Urea, 2% CHAPS, 0.5% IPG Buffer (GE Healthcare), 25 mM DTE and bromophenol blue) were placed in a strip holder with a strip of gel apposed just above. Immobilin drystrip (GE Healthcare) placed in the gel constituted a pH gradient in which proteins migrated in function of their isoelectric point. Before migration, cover fluid (GE Healthcare) was placed on the top of strip holder. The strip holder was then placed on IPGPhor electrodes (GE Healthcare) for a 40,000 Vxh migration. At the end of the migration, the strip holder was stored in a freezer at -80°C prior to the next migration in the second dimension.

Separation in the second dimension: Strips of gel were pre-incubated for 15 min in a buffer permitting the reduction of proteins (50 mM Tris HCl pH 8.8; 6 M Urea; 30% Glycerol; 2% SDS; 65 mM DTE; bromophenol blue) followed by 15 min in a buffer permitting the alkylation of proteins (50 mM Tris HCl pH 8.8; 6M Urea; 30% Glycerol; 2% SDS; 135 mM iodoacetamide, bromophenol blue). The strips were deposited on the top of a polyacrylamide gel concentrated at 12.5% in which protein separation was performed according to their molecular mass. Migration was stopped when 600 Vxh were reached. The electrophoretic buffer used was: 25 mM Tris; 192 mM Glycine; 0.1% SDS.

Mass spectrometry-based protein identification. Proteins were identified as described previously (Couderc, Bollard et al. 2015). Briefly, 2D-PAGE spots of interest were excised and proteins in-gel digested using trypsin. Resulting peptides were analyzed by tandem mass spectrometry (4800 MALDI-TOF/TOF, AB Sciex) and identified using Mascot (Matrix Science) and SwissProt and TrEMBL databases (Homo sapiens taxonomy).

MAb reactivity to K8 peptides by ELISA or Epitope mapping. The K8 peptides, coupled or not with BSA at the SH group of the C-terminal cysteine, were immobilized (0.5 µg/mL or 1 µg/mL, respectively) at the bottom of the wells of a 96-well plate overnight at RT. Unbound peptides were washed away with purified water supplemented with Tween. The wells were then saturated with a saline phosphate buffer solution (PBS) containing 5% BSA for 2 h at RT. The plates were then washed away with purified water supplemented with Tween. MAb binding to the coated peptides was tested at different MAb concentrations following an incubation time of 2 h at RT in PBS containing 1% BSA. Unbound MAbs were washed away with purified water supplemented with Tween. Bound MAbs were then detected using a Goat anti-mouse IgG secondary antibody coupled to horse radish peroxidase (HRP) (Biorad, France). The absorbance was measured at 450 nm and corrected at 630 nm. Direct binding assay: The BSA unconjugated peptides 1, 5 to 28 of human K8 were immobilized at the bottom of a 96-well plate. Different MAb concentrations (from 10 µg/mL onwards with 1:10 dilution increments) were incubated for 2 h at RT alone or with each peptide tested.

Real-time analysis of invasion by X-CELLigence system (ACEA Bioscience). The real-time measurement instrument xCELLigence (ACEA Biosciences™) is based on the impedance value which is proportional to the fixation of invasive cells on microelectrodes. The system used to evaluate the invasive capacities of cells is the RTCA DP system. This system uses CIM plates containing 16 wells. Wells are formed by a lower and an upper chamber placed at 37°C and 5% CO_2. The upper chamber communicates with the lower chamber through 8 µM in diameter pores which are above the 10 micro-electrodes. When evaluating Isreco-1 invasive capacities, the lower chamber contained culture medium supplemented with 10% FBS and the upper chamber was deprived of FBS (SF medium). The bottom of this upper chamber was previously covered with a layer of matrigel (matrigel™, BD biosciences). Isreco-1 cells (20,000 cells) were placed in the upper chamber with or

without the anti-K8 antibodies (M20, D-A10 or D-D6) at 50 μg/mL for testing the effects of these antibodies, or were placed with or without purified plasminogen (Abcam) at different concentrations (500, 50 or 5 nM) or with purified plasminogen at 50 nM and D-A10 Fab MAb or isotypic control Fab MAb at 160 nM (25 μg/mL) when competition for eK8 binding was analyzed. For experiments with purified plasminogen, cells were previously grown for 24 h in SF medium. During the invasion process, cells are attracted by the FBS gradient established between the lower and upper chamber, degrade the matrigel allowing them to migrate through the pores and enter into contact with the micro-electrodes of the lower chamber. The impedance generated by cell contact is measured every 15 min and is recorded by a computer linked to the RTCA DP system. This process was evaluated over 70 h for Isreco-1 cells and was calculated by integrating the slopes of the mean curves of triplicate experiments ± SEM.

Western blot analysis. In Western blot (WB) experiments, 5-15 μg of proteins from Isreco-1 and HCT116 cell lysates were separated by SDS-PAGE and transferred onto a nitrocellulose membrane. The membrane was saturated with a solution of TBS-T (20 mM Tris-HCl, pH 7.0, 130 mM NaCl, 0.1% Tween 20) containing 5% milk, then incubated with various antibodies diluted in TBS-T solution containing 2.5% milk. The anti-K8 M20 (50 μg/mL), 1E8 (2 μg/mL), D-A10, D-D6 and 2G11 (1 μg/mL), anti-casp3 Alexis (1:250), anti-Ku80 (1:2,500), anti-histone H3 (1:2,000), anti-Na+K+ATPase (1:5,000) antibodies were incubated for 1 h at RT with the membrane. The primary antibodies were detected with anti-mouse or anti-rabbit secondary antibodies coupled to HRP.

WB competition assay: For the competition experiments, D-A10 orD-D6 MAbs (1 μg/mL) were pre-incubated for 2 h at RT with peptide 1 or peptide 2 with a molecular ratio of 1:100 (one antibody molecule per 100 peptide molecules) in a TBS-T solution. The antibody-peptide mixture was then brought into contact with the membranes in a solution of TBS-T containing 2.5% milk for 1 h followed by a detection using an anti-mouse secondary antibody coupled to HRP.

Native gel: Non-denaturing loading buffer was added to protein lysates, and proteins were then separated on gels without SDS. A cold electrophoretic migration was performed with anode buffer (25 mM imidazol) and cathode buffer (50 mM tricine, 7.5 mM imidazol, 0.05% deoxycholate, 0.01% DDM). Protein transfer and immunoblotting were conducted as described above.

Expression of GFP-K8 construct by transfection. 1.10^6 cells of HCT116 cells were seeded onto 6-well plates 24 h before transfection. Then 1 μg of expression plasmid containing the full length human K8 cDNA sequence sequence coding for GFP-K8 (Loranger et al, Exp Cell Research 2006) were added to OptiMEM medium (Gibco) in presence of X-tremeGENE HP DNA Transfection Reagent (Roche) with a ratio of transfection reagent/plasmid of 3:1. Cells were incubated with the mixed preparation for 24 h or 48 h at 37 °C and 5% CO_2. Cells cultured on glass-coverslips were then fixed with 4% paraformaldehyde for immunofluorescence analysis or were scrapped for WB analysis as described above.

***In vivo* experiments.** All *in vivo* experiments were done in agreement with the Institutional Animal Care.

Effect of anti-K8 antibodies on Isreco-1 colorectal cancer cells

Isreco-1 cells (10.10^6) were injected subcutaneously into 12 SCID CB17 mice. After 15 days, the tumors reached a volume between 100 and 150 mm^3. At this stage, mice were divided into four groups of three mice. Each group received or not a specific treatment by intraperitoneal injection performed at the opposite site of the tumor. Once a week during 4 weeks (days 15, 22, 29 and 36), mice received or not an injection of 30 mg/kg of M20 antibody, or of D-A10 or D-D6 MAbs. The average tumor volume of each mouse group is represented on the graphs with a standard deviation corresponding to three mice per group.

Dose effect of D-A10 MAb on Isreco-1 colorectal cancer cells

Isreco-1 cells (10.10^6) were injected subcutaneously into 70 SCID CB17 mice. After 10 days tumors reached a volume between 100 and 150 mm3. At this time mice were divided into four groups of ten mice. Each group received or not a specific treatment by intraperitoneal injection performed at the opposite site of the tumor. Once a week during 4 weeks (days 12, 19, 26 and 33), mice received or not an injection of 0,3 mg/kg, 1 mg/kg, 3 mg/kg, or 10 mg/kg of D-A10 Mab. For each mouse, the

tumor volume was measured twice weekly throughout the experiment. The average tumor volume of each mice group is represented on the graph with a standard deviation corresponding to three mice per group.

Effect of anti-K8 antibodies on HCT116 colorectal cancer cells

The HCT116 cells (10x106) were injected subcutaneously in 12 SCID CB17 mice. After 6 days, the tumor reached a volume between 100 and 150 mm3. At this time, mice were divided in four groups of three mice. Each group received or not a specific treatment by intraperitoneal injection performed at the opposite site of the tumor. Once a week during 3 weeks (days 6, 13 and 20), mice received or not an injection of 30 mg/kg of the antibody anti-K8 (M20 antibody, DA10 or D-D6 MAbs). For each mouse, the tumor volume was measured twice weekly throughout the experiment. The average volume of each group of mice is shown in the graph with a standard deviation equal to three mice per group.

Dose effect of D-A10 MAb on HCT116 colorectal cancer cells

The HCT116 cells (10x106) were injected subcutaneously in 18 SCID CB17 mice. After 10 days the tumor reached a volume between 100 and 150 mm3. At this time, the mice were divided into four groups of three mice. Each group received or not a specific treatment by intraperitoneal injection at the opposite site of the tumor. Once a week for three weeks (days 10, 17, 24 and 32), the mice were treated or not with 1 mg/kg, 3 mg/kg or 10 mg/kg of D-A10 Mab. For each mouse, the tumor volume was measured twice weekly throughout the experiment. The average volume of each group of mice is shown in the graph with a standard deviation equal to three mice per group.

Immunohistochemistry analysis. Tissues were isolated from Isreco-1 xenograft tumors of mice treated or not with MAbs at 30 mg/kg. For staining, tissue samples were fixed in 10% buffered formalin and embedded in paraffin. 4 µm-thick tissue sections of formalin-fixed, paraffin-embedded tissues were prepared according to conventional procedures. Immunohistochemistry was performed on an automated immunostainer (Ventana Discovery XT, Roche, Meylan, France) using DABmap Kit according to the manufacturer's instructions. After retrieval procedures with buffer Cell Conditioning 1 (CC1), sections were incubated with rabbit monoclonal anti-cleaved caspase 3 antibody (diluted 1:200, clone 5A1E, Cell signaling) or mouse monoclonal anti-Ki67 antibody (diluted 1:50, clone Mib1, DAKO Ki67) for 1 h. Staining was visualized with DAB solution with 3,3′-diaminobenzidine as a chromogenic substrate. Finally, the sections were counterstained with Gill's hematoxylin. Quantification of the number of caspase 3 activated-positive (Casp3A) cells per mm^2 and of Ki67-positive cells compared to total cells was performed using the Histolab software coupled to a camera after microscopic visualization. For each tumor, counting of the number of Casp3A- and Ki67-positive cells was conducted on a surface of 2 mm^2. Necrotic areas of the tumor and fibrous capsules were excluded from the analysis. The number of Casp3A- and Ki67-positive cells is represented with a standard deviation corresponding to three mice per group, or ten mice per group for Casp3A only.

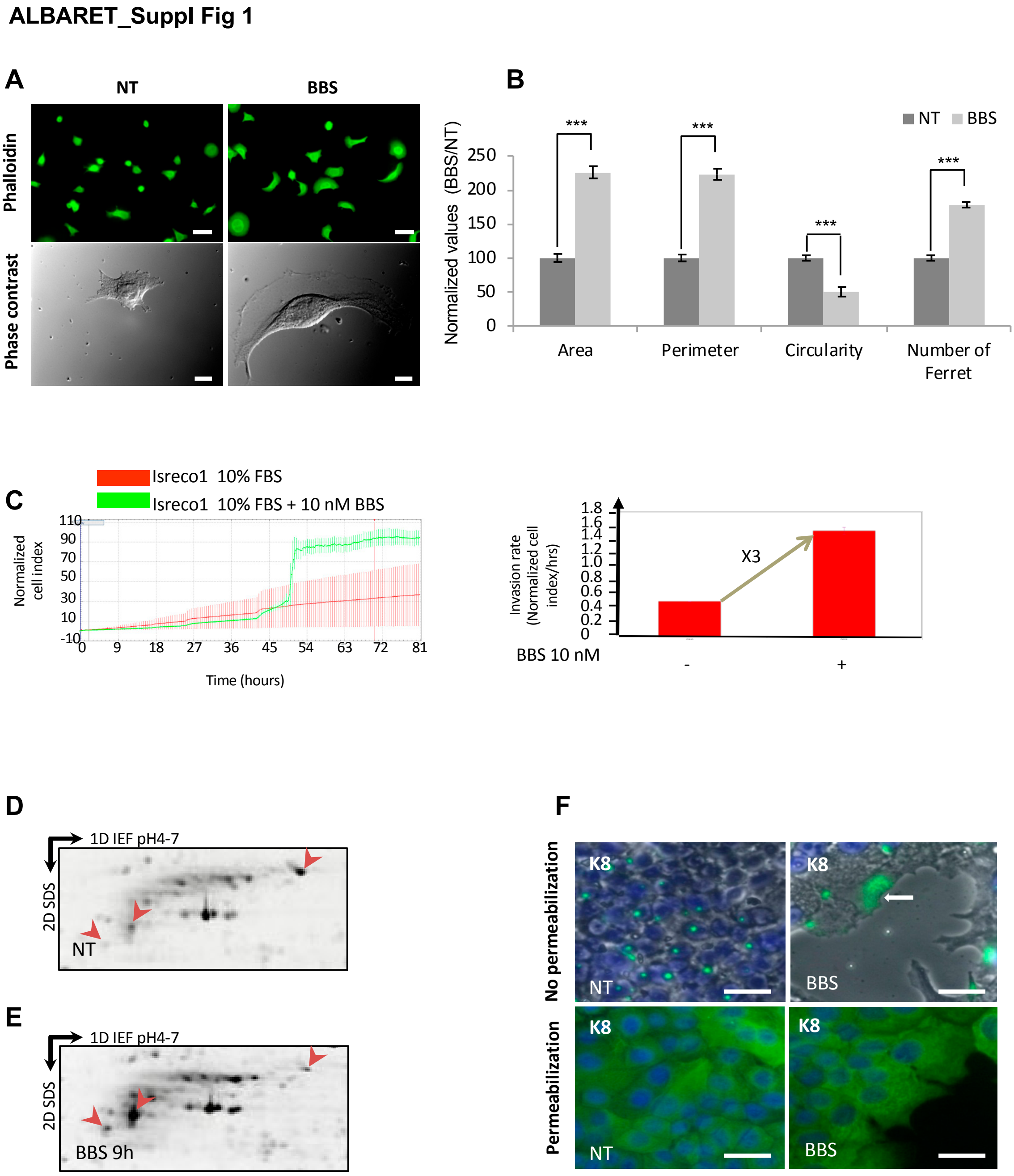

Figure S1. K8 is accumulated in a cellular model of colorectal cancer cell invasion. (**A**) Fluorescent micrograph of phalloidin staining (top panel, Scale bar = 50 µm) and light microscopy (bottom panel, Scale bar = 10 µm) of Isreco-1 cells treated with the bombesin (BBS) peptide. (**B**) Quantification of cellular parameters characteristic of spreading conducted on phalloidin-stained cells. A 220% increase in the area and perimeter of cells, as well as a 50% decrease in circularity were observed. The number of Ferret, which represents the two most distant points in a cell, also increased significantly. (C) Real-time analysis using the X-CELLigence system of Isreco-1 cell invasion after treatment with BBS. Histogram representing (panel of left) the integration of the mean invasion curve slopes (triplicate of three wells per condition ± SEM) over 70 h, calculated using the RTCA software®. (**D**–E) Autoradiography of 2.D gels of Isreco-1 cytoplasmic fraction after short metabolic labeling with Sulfur S^{35} and treatment (**E**) or not (**D**) with BBS for 9 h. Proteins with the most significant variation in

synthesis after BBS treatment are indicated with red arrows. (**F**) Immunofluorescence on non-permeabilized (upper panel) and permeabilized using Tween 20 (lower panel) Isreco-1 cells treated or not with bombesin (BBS). Plasma membrane and intracellular localization of K8 was highlighted using the M20 anti-K8 antibody (green signal). Scale bar = 50 μm.

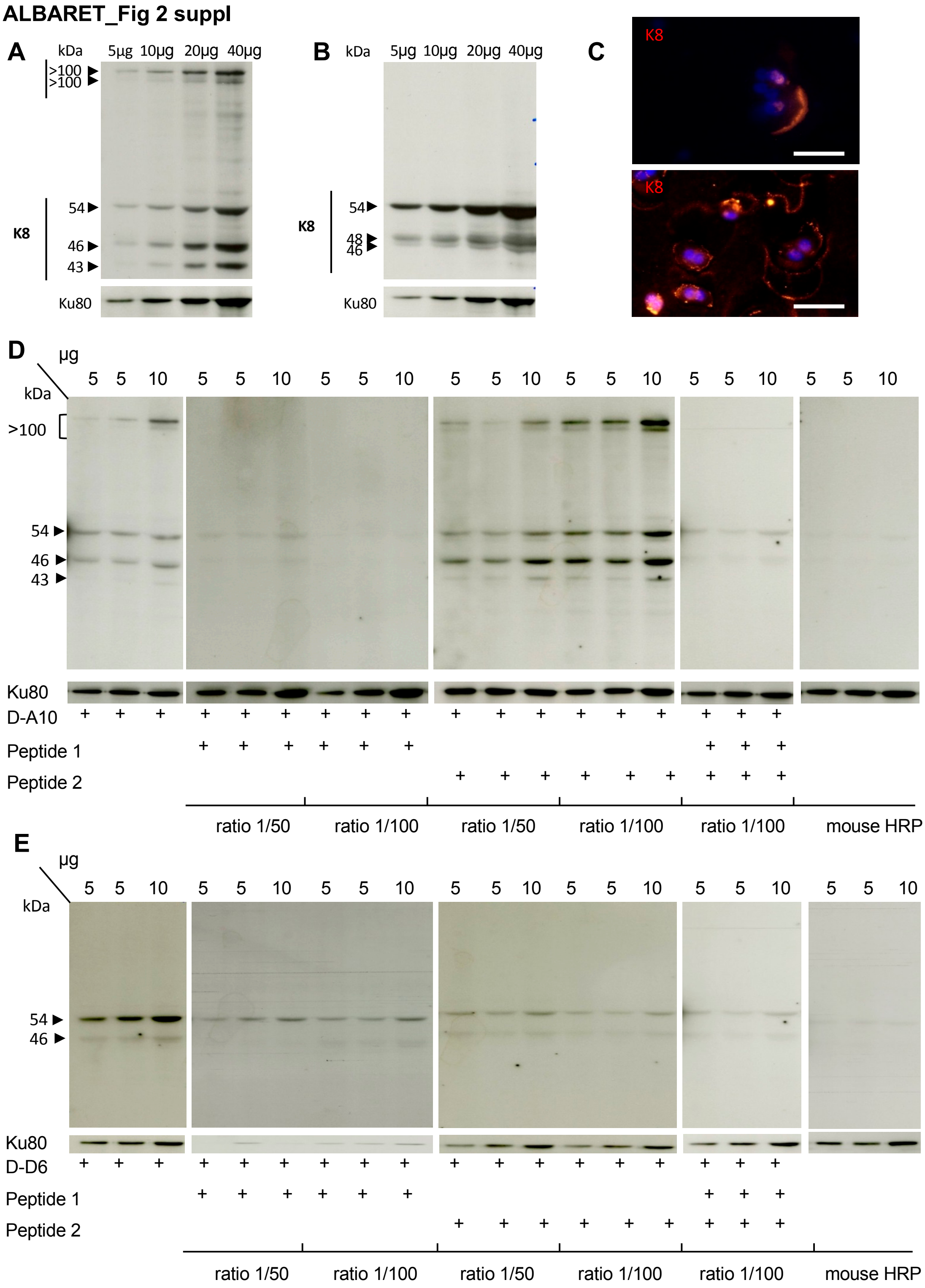

Figure S2. Characterization of D-A10 and D-D6 MAb properties. (**A**–**B**) Western blot (WB) analysis of K8 isoforms detected by D-A10 (A) or D-D6 (**B**) MAbs in Isreco-1 cell lysates. (**C**) Immunofluorescence analysis of externalized K8 (red signal) detected by D-A10 (upper panel) or D-D6 (lower panel) MAbs on non-permeabilized Isreco-1 cells. Hoechst dye was used to counterstain

the nucleus (blue signal). Scale bar = 50 µm. (**D**–**E**) WB competition experiments were performed with 5 or 10 µg of proteins from Isreco-1 cell lysate. WB analysis of K8 isoforms detected using the D-A10 MAb (D) or D-D6 MAb (**E**) after incubation or not with K8 peptide 1 and/or peptide 2 with a molecular ratio of 50 or 100 moles of peptide for 1 mole of MAb (see Supplemental information).

ALBARET_Fig 3 suppl

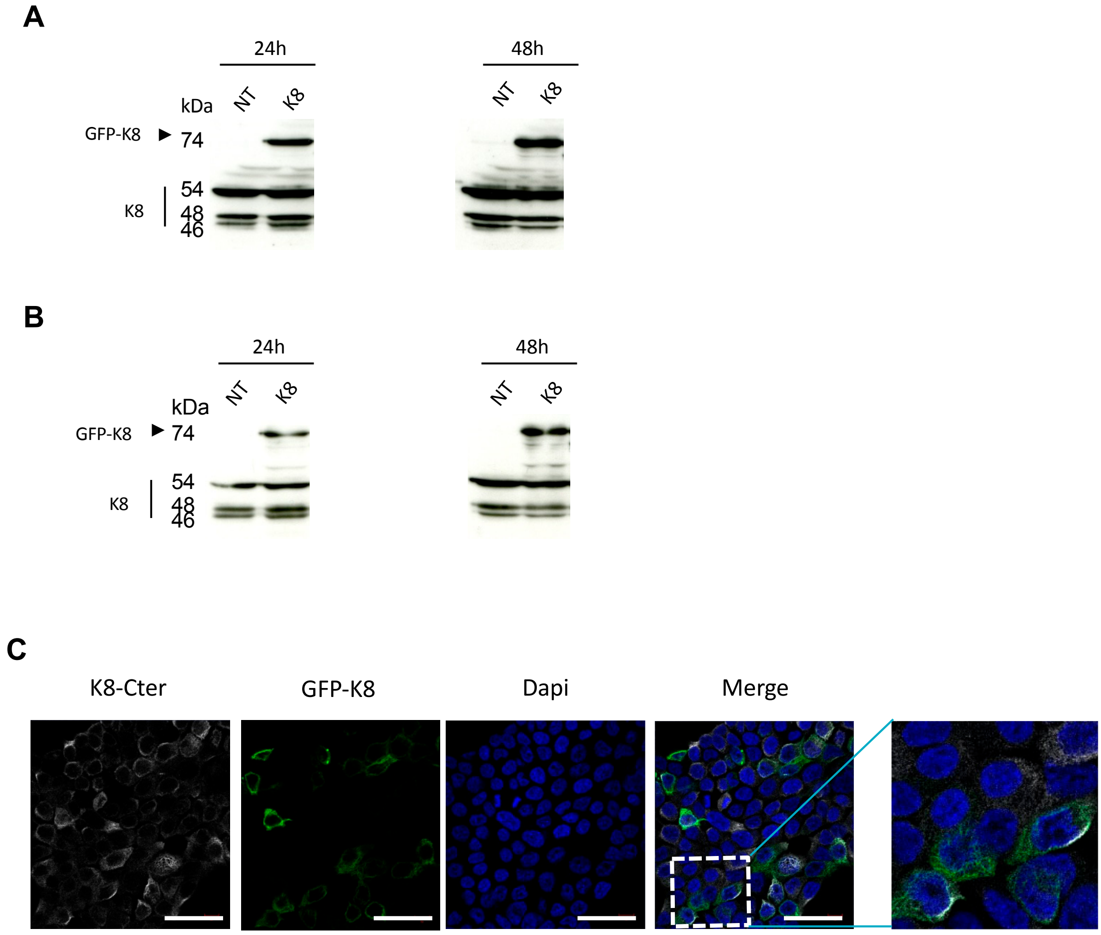

Figure S3. Expression of GFP-K8 constructs. (**A**–**B**) Transfection of HCT116 cells with GFP-K8 constructs for 24 h (**A**) or 48 h (**B**) and Western blot analysis of K8 accumulation using M20 and 1E8 antibodies. (**C**) Transfection of HCT116 cells with the GFP-K8 construct and confocal microscopy. Direct visualization of GFP fluorescence (green signal). The C-terminal part of K8 was highlighted by IF analysis on non-permeabilized cells using the anti-C ter K8 antibody (white signal). Co-localization of green and white signals is presented on the enlarged view of the merged image (right panel). Scale bar = 50 µm.

ALBARET_Fig 4 suppl

A

#	Template	Alignment Coverage	3D Model	Confidence	% i.d.	Template Information
1	c1ciiA_	Alignment	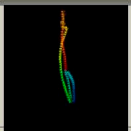	99.6	11	**PDB header:**transmembrane protein **Chain:** A: **PDB Molecule:**colicin ia; **PDBTitle:** colicin ia
2	c3s4rB_	Alignment	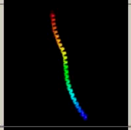	99.6	39	**PDB header:**structural protein **Chain:** B: **PDB Molecule:**vimentin; **PDBTitle:** crystal structure of vimentin coil1a/1b fragment with a stabilizing2 mutation
3	c3tnuA_	Alignment	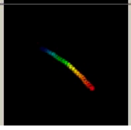	99.5	33	**PDB header:**cytosolic protein **Chain:** A: **PDB Molecule:**keratin, type i cytoskeletal 14; **PDBTitle:** heterocomplex of coil 2b domains of human intermediate filament2 proteins, keratin 5 (krt5) and keratin 14 (krt14)

B

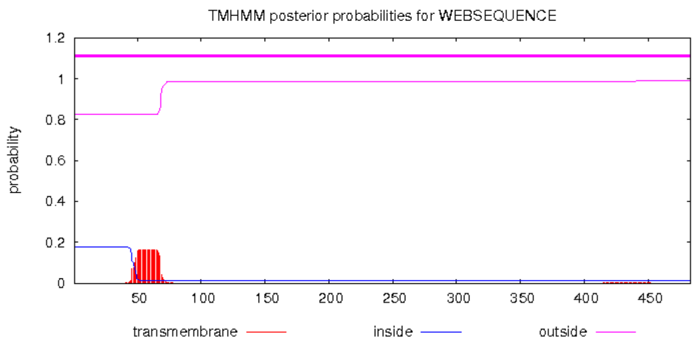

C

10 20 30 40 50 60 70 80 90
MSIRVTQKSYKVSTSGPRAFSSRSYTSGPGSRISSSSFSRVGSSNFRGGLGGGYGGASGMGGITAVTVNQSLLSPLVLEVDPNIQAVRTQ

——— non canonical α helix

Figure S4. Analysis of K8 secondary structure and primary sequence. (**A**) Comparison of the secondary structure of K8 and different proteins. Colicin-ia presents the strongest homology with K8. (**B**) Transmembrane domain prediction using the TMHMM web interface. (**C**) Primary sequence of K8 from amino-acid (aa) 1 to 90. Two hypothetical non-canonical α-helices are underlined between aa 45 to 63 and 68 to 88.

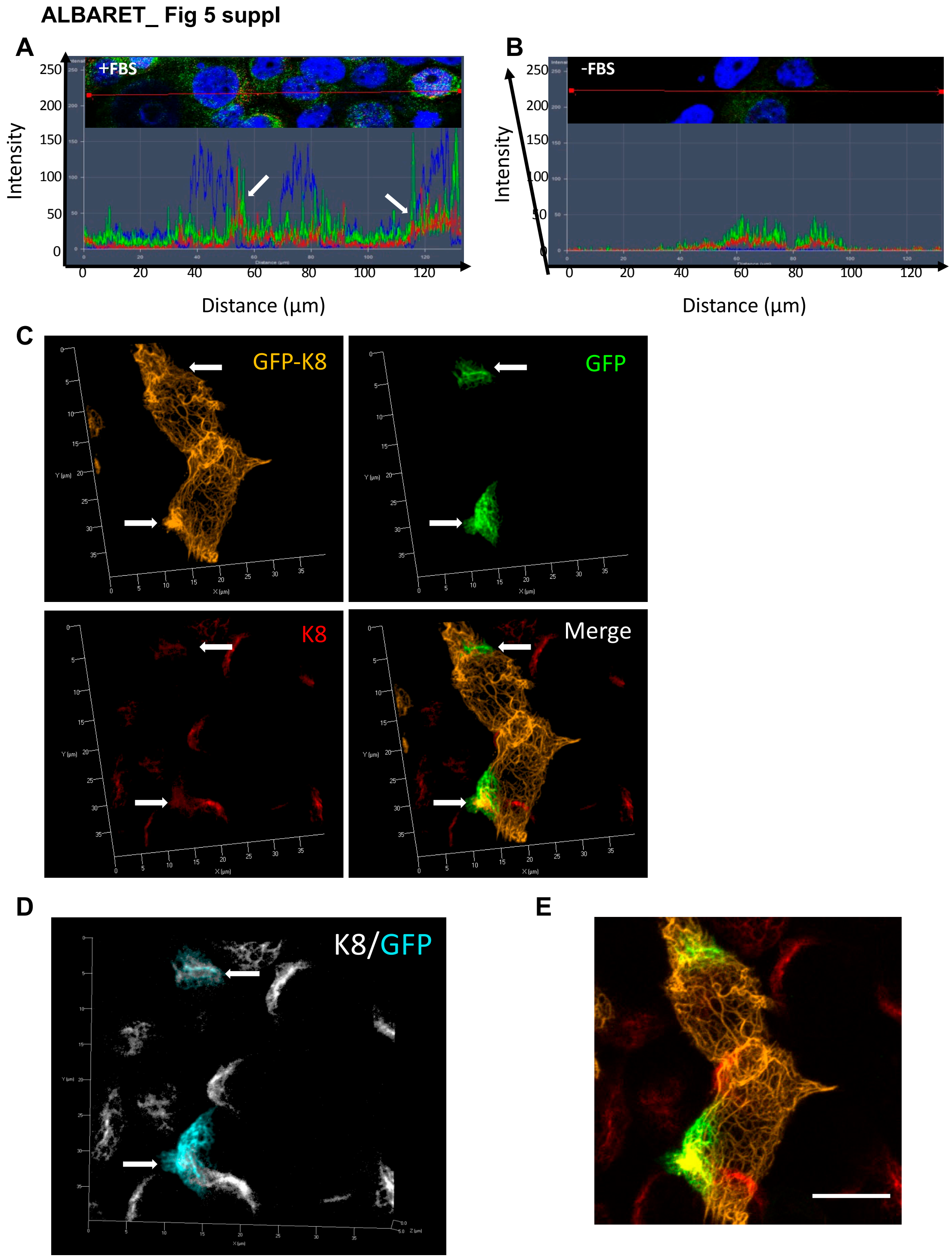

Figure S5. Confocal analysis of K8-Plg and K8-GFP co-localization by immunofluorescence (IF). (**A**–**B**) IF analysis by confocal microscopy of K8 (N-ter antibody, green signal) and plasminogen (anti-Plg antibody, red signal) localization on non-permeabilized Isreco-1 cells cultured in the presence (**A**) or absence of FBS (**B**). Quantification of the fluorescent signals intensity (K8 and Plg) all along a cross optic section (red line). K8 and Plg co-localization in Isreco-1 cells in presence of FBS is indicated by

white arrows. Hoechst dye was used to counterstain the nucleus. (**C**–D) Transfection of HCT116 cells with the GFP-K8 construct and confocal microscopy with 3D reconstruction. IF analysis on non-permeabilized HCT116 cells showing individual channel (**C**) of GFP-K8 fluorescence (Yellow), of anti-GFP signal (green signal), endogenous K8 alone (M20 antibody, red signal), and the merged image (same as in Figure 5A). (**D**) shows overlay of anti-K8 (white) and anti-GFP (cyan) signals. Co-localization of endogenous K8 and GFP is indicated with white arrows in panel D and reported in panel C. (**E**) z-projection of all planes of the 3D-stack shown in Figure 5A (maximum intensity projection). Scale bar = 10 μm.

References

1. Van Muijen GN, Ruiter DJ, Warnaar SO. Coexpression of intermediate filament polypeptides in human fetal and adult tissues. Lab Invest 1987;57:359-69.
2. Waseem A, Karsten U, Leigh IM, et al. Conformational changes in the rod domain of human keratin 8 following heterotypic association with keratin 18 and its implication for filament stability. Biochemistry 2004;43:1283-95.
3. Belin, S., A. Beghin, E. Solano-Gonzalez, L. Bezin, S. Brunet-Manquat, J. Textoris, A. C. Prats, H. C. Mertani, C. Dumontet and J. J. Diaz (2009). "Dysregulation of ribosome biogenesis and translational capacity is associated with tumor progression of human breast cancer cells." PLoS One **4**(9): e7147.
4. Couderc, C., J. Bollard, Y. Coute, P. Massoma, G. Poncet, F. Lepinasse, V. Hervieu, N. Gadot, J. C. Sanchez, J. Y. Scoazec, J. J. Diaz and C. Roche (2015). "Mechanisms of local invasion in enteroendocrine tumors: identification of novel candidate cytoskeleton-associated proteins in an experimental mouse model by a proteomic approach and validation in human tumors." Mol Cell Endocrinol **399**: 154-163.
5. Johnson, A. M., P. J. McNamara, S. H. Neoh, P. J. McDonald and H. Zola (1981). "Hybridomas secreting monoclonal antibody to Toxoplasma gondii." Aust J Exp Biol Med Sci **59**(Pt 3): 303-306.
